# Evidence That Marine Reserves Enhance Resilience to Climatic Impacts

**DOI:** 10.1371/journal.pone.0040832

**Published:** 2012-07-18

**Authors:** Fiorenza Micheli, Andrea Saenz-Arroyo, Ashley Greenley, Leonardo Vazquez, Jose Antonio Espinoza Montes, Marisa Rossetto, Giulio A. De Leo

**Affiliations:** 1 Hopkins Marine Station, Stanford University, Pacific Grove, California, United States of America; 2 Comunidad y Biodiversidad A.C., Colonia Hipódromo Condesa, México DF, México; 3 Sociedad Cooperativa de Produccion Pesquera Buzos y Pescadores, Isla Natividad, Baja California Sur, México; 4 Environmental Science Department, University of Parma, Parma, Italy; University of California Davis, United States of America

## Abstract

Establishment of marine protected areas, including fully protected marine reserves, is one of the few management tools available for local communities to combat the deleterious effect of large scale environmental impacts, including global climate change, on ocean ecosystems. Despite the common hope that reserves play this role, empirical evidence of the effectiveness of local protection against global problems is lacking. Here we show that marine reserves increase the resilience of marine populations to a mass mortality event possibly caused by climate-driven hypoxia. Despite high and widespread adult mortality of benthic invertebrates in Baja California, Mexico, that affected populations both within and outside marine reserves, juvenile replenishment of the species that supports local economies, the pink abalone *Haliotis corrugata*, remained stable within reserves because of large body size and high egg production of the protected adults. Thus, local protection provided resilience through greater resistance and faster recovery of protected populations. Moreover, this benefit extended to adjacent unprotected areas through larval spillover across the edges of the reserves. While climate change mitigation is being debated, coastal communities have few tools to slow down negative impacts of global environmental shifts. These results show that marine protected areas can provide such protection.

## Introduction

Marine ecosystems worldwide are affected by a suite of stressors that combine to degrade whole ecosystems and the many services they provide [1]–[5]. Many stressors, including impacts from climate change, cannot be removed at local scales. However, enhanced local resilience - the ability of populations and ecosystems to absorb disturbance while retaining their function and provision of ecosystem services - may help combat the impacts of these major disturbances [6]–[8]. Thus, enhancement of resilience through the removal or amelioration of local disturbance may provide the best opportunity for local communities to respond to global climate change [9].

A means of removing or ameliorating local disturbance is through the establishment of marine reserves - areas of the ocean that are fully protected from extractive activities. Marine reserves can promote the recovery of overexploited populations, enhance fisheries yields through spillover across reserve boundaries, restore species interactions and food web dynamics, empower local communities, and provide additional income from fishing and tourism [10]–[17]. However, in contrast to this demonstration of benefits, some studies have highlighted continued climatic impacts in the presence of marine reserves [3]–[4], [18]–[19]. As a result, active discussion is ongoing on whether reserves can increase the resilience of marine populations and ecosystems [4], [20], and what components of resilience – resistance to disturbance, or recovery rates - are most effective at moderating local climate change impacts [3]. Despite the common hope that reserves play this role, experiments that demonstrate the effectiveness of local protection against global problems are lacking. Thus, some authors have argued that the expectation that a reduction of local stressors, such as fishing, provides increased resilience to climate change may be incorrect [3]–[4]. An alternative prediction is that local stressors may select for resistant species and individuals, thereby decreasing the impacts of climatic disturbance, if tolerance to a non-climatic disturbance is correlated with tolerance to climatic impacts [3]. Here, we asked whether marine reserves increase the resilience of marine populations to widespread mortality likely caused by climate-driven hypoxia.

We focused on abalones, *Haliotis* spp., because of their high commercial value and depleted status. Abalones are large herbivorous mollusks that have supported highly valuable coastal fisheries in Canada, the USA, Mexico, South Africa, Australia, and Japan since the mid 1800s [21]–[23]. Most of these fisheries have collapsed, failing to recover thereafter [22]–[23]. In California, USA, *Haliotis* spp. (5 species) total catches dropped from a peak of 24,000 tons to 115 tons by 1995, culminating in the 1997 closure of all commercial and sport fishing south of San Francisco [23]. Two species, the white (*H. sorenseni*) and black (*H. cracherodii*) abalone, were included in the US Endangered Species List, and four additional species are under evaluation. In contrast, Baja California, Mexico, still has commercial pink (*H. corrugata*) and green (*H. fulgens*) abalone fisheries, worth approx. US $ 20,000,000/year. These fisheries are the main source of income for coastal communities along the Pacific coast of the peninsula. However, recent total catches of ∼500 tons are six times lower than catches through the 1960s-1970s and ten times lower than the maximum recorded catches in the early 1950s [24].

**Figure 1:**
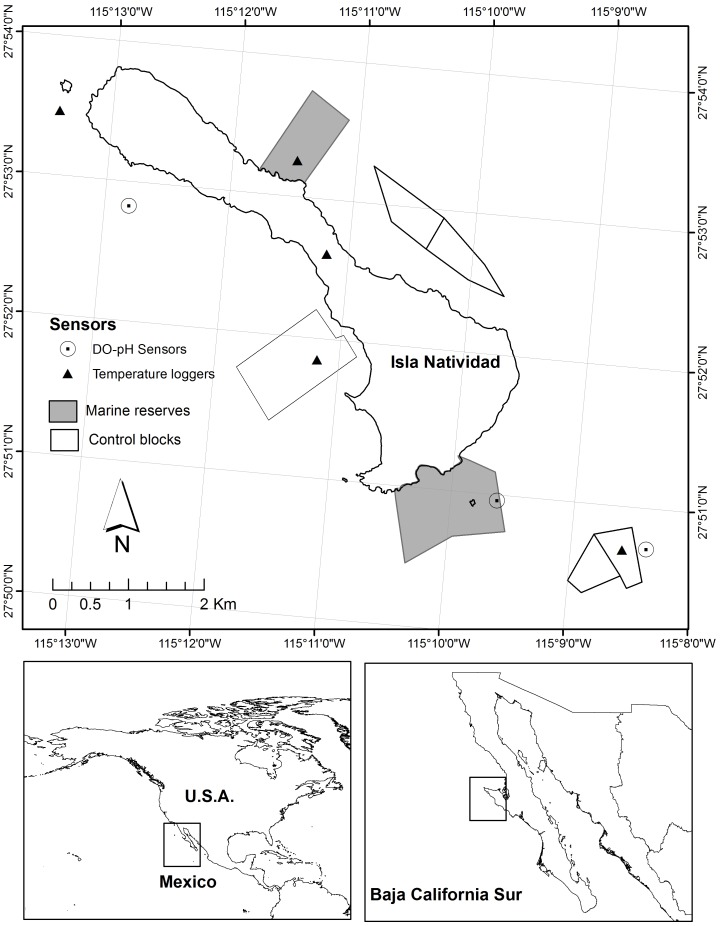
Map of the study area, in Isla Natividad (top panel), Baja California, Mexico (bottom panels), showing the location of the no-take (marine reserves) and fished, reference areas (control blocks). The location of oceanographic sensors (temperature, DO and pH sensors and loggers) is also shown.

In response to continued decline, in 2006 the fishing cooperative of Isla Natividad, along the Pacific coast of Baja California, established two marine reserves (http://www.cobi.org.mx/?pag=r-pbc-isla-natividad&idioma=eng) excluding all take from 8% of the fishing grounds surrounding the island (Fig. 1). Reserves were established voluntarily by the fishing cooperative, with no-take regulations enforced locally by the cooperative itself. Selection of the location and size of reserves was done by the members and staff of the fishing cooperative based on biological (high past productivity of the areas, as measured by catches of abalones and other target benthic invertebrates) and economic (the estimated lost income associated with the establishment of the no-take areas) considerations. The goal of reserves was to recover depleted abalone populations and fisheries through larval spillover from reserves to fishing grounds.

Isla Natividad is located in an area of intense upwelling and recently experienced shoaling of hypoxic waters similar to climate-driven events documented in other areas of the California current [25]–[27]. In spring 2009, fishermen reported unusually high mortality of abalones and other benthic invertebrates (e.g., sea urchins, *Strongylocentrotus* spp., turban snail, *Megastraea* spp., and key-hole limpets, *Megathura crenulata*) probably due to an unmonitored hypoxia event at sites in Isla Natividad. Fishermen reported that animals were weak and were easily detached from the substrate, and that benthic and demersal fishes usually found close to the seafloor were aggregating at the water surface, consistent with the presence of bottom hypoxia and the shoaling behavior of fishes we observed the following year, when we documented a hypoxic event at Isla Natividad (see *Results*). Moreover, in summer 2009, DO concentrations as low as 0.9 mg/L were measured at sites 200 km south of Isla Natividad (off La Bocana, 26° 46′ N, 113° 43′ W, also within the Vizcaino region of Baja California Sur; D. Aguilar, unpublished data). Annual biomass estimates conducted jointly by the fishing cooperative and the regional fisheries agency indicated that mass mortality caused an estimated 75% reduction of abalone biomass within the fishing grounds and 50% within the marine reserves. Less intense mortality was also associated with the 2010 hypoxic event we monitored.

To assess the efficacy of reserves in recovering abalone populations from fishing impacts, between 2006–2010 we monitored the abundance, size structure, reproductive output, and post-larval recruitment of pink abalones (*Haliotis corrugata*) within the reserves and in adjacent fished areas with similar habitat characteristics. Our data precede the 2009 mortality event, allowing us an unprecedented view of its demographic effects, both within reserves and in fished areas. Prior to 2009, fishers had not witnessed sudden and widespread benthic invertebrate mortality.

## Results

In 2010, physical monitoring conducted at three locations around Isla Natividad (Fig. 1) revealed prolonged periods (up to 21 consecutive hours) when dissolved oxygen (DO) concentrations were at or below 2 mg/L (Fig. S1), the mean lethal threshold for mortality across 206 marine organisms exposed to low DO in the laboratory [28]. Moreover, during the same time, DO was at or below 4.6 mg/L for periods of up to 23 consecutive days (Fig. S1), longer than the mean lethal time of 11.1 days reported in the study cited above [28]. In 90% percent of 872 experiments, lethal DO concentrations were below 4.6 mg/L [28].

**Figure 2:**
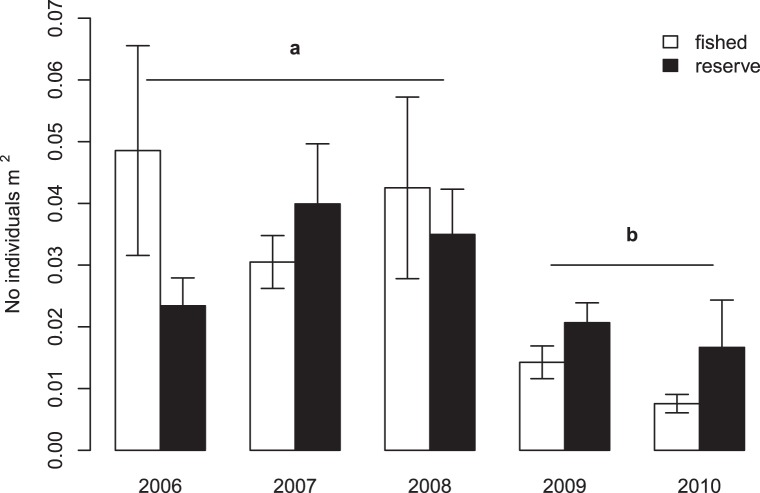
Abalone densities within reserves and fished areas in 2006–2010. Yearly averages (+1SE, N = 11–30 transects per treatment combination) overlain by the same letter (a or b) are not significantly different at α = 0.05 in post-hoc comparisons.

A combination of fishing and possibly hypoxia resulted in significant abalone decline between 2006–2010 (Fig. 2). Monitoring data show a significant decrease of pink abalone densities in 2009 and 2010 compared to 2006–2008 (ANCOVA, year: *P* = 0.001; Table S1), coinciding with the high mortality event (Fig. 2). The effect of protection on adult densities was overall not significant (Table S1), as expected for an external environmental perturbation such as a hypoxic event affecting animals both within and outside reserves. Moreover, the protection by year interaction term was also not significant, indicating no statistically significant temporal variation in mortality between reserves and fished sites (Table S1). However, decline tended to be more pronounced at fished sites, revealing a negative impact of fishing: a six-fold decrease from 2006 to 2010 (avg. 2006 = 0.049±0.081SE individuals/m^2^, avg. 2010 = 0.007±0.013SE individuals/m^2^; Fig. 2), compared to reserves where 2010 densities were, on average, twice what was estimated at fished sites and similar to values estimated in 2006 (avg. 2006 = 0.023±0.029SE individuals/m^2^, avg. 2010 = 0.017±0.053SE individuals/m^2^; Fig. 2).

Average abalone densities within reserves were 1.7 times lower in 2009–2010, after the mortality event, compared to 2006–2008, indicating that protected populations had been negatively affected. However, such decline was greater outside reserves, with densities in 2009–2010 3.7 times lower than during the previous three years (averaged over these time periods). Thus, as expected, combined fishing and natural mortality had a greater impact on fished compared to protected populations that were affected only by natural mortality. As a result of these different trends, abalone densities were similar between reserves and fished areas in 2006–2008 (reserves: 0.03±0.004 individuals/m^2^; fished: 0.04±0.007 individuals/m^2^), but were double in reserves than fished areas in 2009–2010, after the mortality event (reserves: 0.02±0.004 individuals/m^2^; fished: 0.01±0.001 individuals/m^2^) (Fig. 2).

Following the mortality events, greater proportions (approx. 10%) of large individuals persisted inside reserves, particularly above the minimum legal size for this fishery (14 cm in length; Fig. S2 and Table S2). In 2010, an average of 92% of individuals encountered in field surveys were above the reported size of sexual maturity (10.3 cm in length) [22] and 45% above the minimum legal size within reserves, compared to 81% and 35% in fished areas, respectively (Fig. S2 and Table S2).

**Figure 3:**
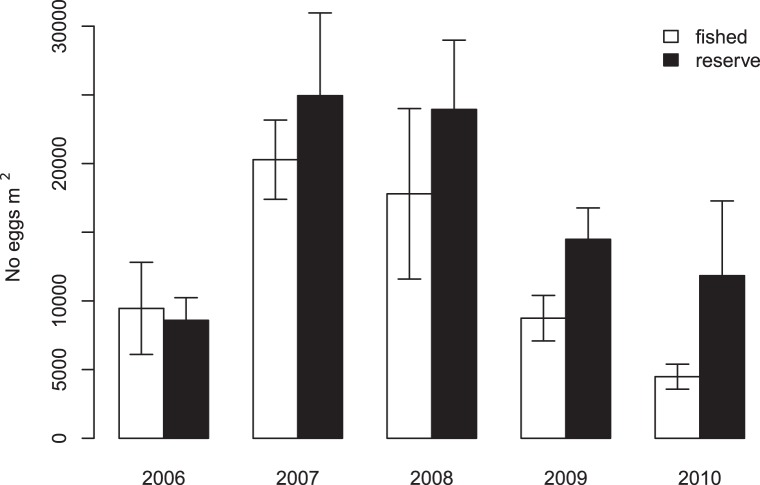
Estimated reproductive output of pink abalones from reserves and fished areas in 2006–2010. Reproductive output is calculated as No. eggs produced · m^−2^ · year^−1^. Error bars are bootstrapped standard deviations (SD).

Combined responses of abalone densities and size structure to protection enhanced the reproductive output of protected populations (Fig. 3). In 2006, when reserves were established, estimated egg production was similar between reserves and fished areas (Fig. 3). The difference in egg production between reserves and fished areas increased in each subsequent year of protection. Following the mortality events, estimated reproductive outputs in the reserve were 1.6 and 2.6 times greater than in fished areas in 2009 and 2010, respectively (Fig. 3; *P*≤0.05 in both years, Table S3). Egg production in 2010 was half what was estimated in 2006 in fished areas, but increased by 40% in reserves, despite high adult mortality (Fig. 3).

**Figure 4:**
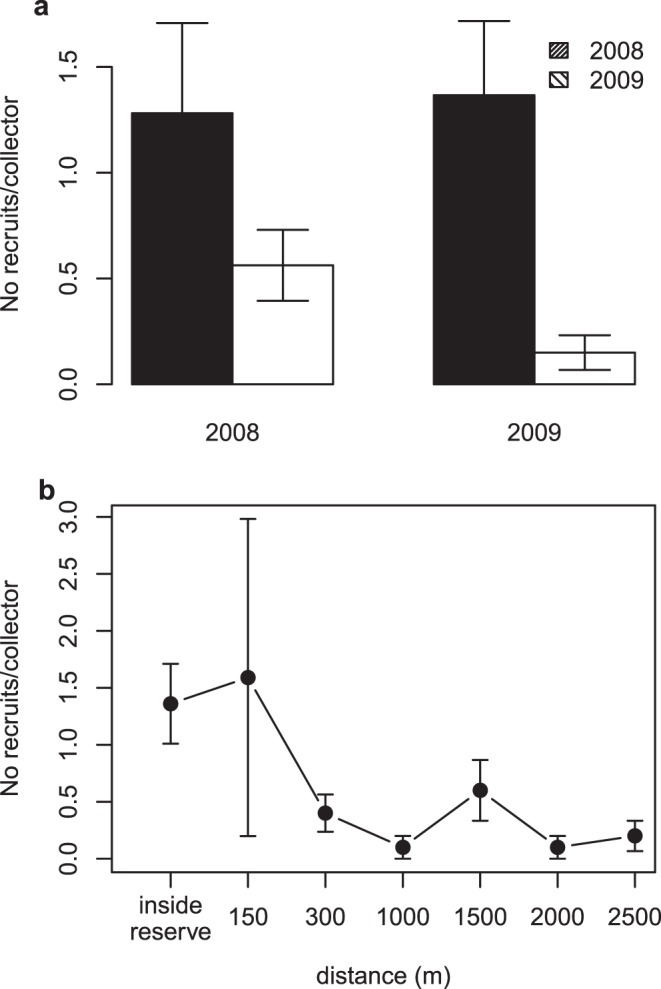
Postlarval recruit abundance within and outside marine reserves. (a) Postlarval recruit abundance (averaged across the recruitment season, ±1SE) within the Punta Prieta reserve and nearby fished area in 2008 and 2009, before and after the mass mortality event of spring 2009; (b) postlarval recruit abundance (averaged across the recruitment season) within the reserve and at varying distances from the reserve edge.

High post-mortality egg production in reserves resulted in significantly greater juvenile recruitment in reserves compared to fished areas (ANOVA: *P*<0.05 in 2008; *P*<0.01 in 2009; Tables S4 and S5). In 2008, recruitment rates were, on average, 2.3 times greater in the reserves than in the fished areas (Fig. 4a). In 2009, after the mortality event, recruitment rates remained stable in the reserve but were 3.8 times lower, on average, than in 2008 in the fished area, and 9.1 times lower than in the reserve (Fig. 4a). Greater recruitment rates were not limited to a single reserve, but were instead documented in both reserves compared to paired fished areas (Table S5).

High recruitment rates were detected at locations inside reserves and within 300 m from the reserve edge, indicative of larval spillover from the reserve to adjacent fished areas (Fig. 4b). Recruitment rates at distances greater than 300 m from the reserves’ edges, except for 1500 m, were significantly lower than within reserves and just outside their edges (Figs. 4b and S3; Table S6; significance of the observed differences in recruitment at increasing distance from the reserve edge was tested with SNK pairwise comparisons, at α = 0.05). Recruitment rates 150, 300 and 1500 m from the reserve edges were highly variable among dates, and not significantly different from recruitment measured within the reserve.

## Discussion

These results show that protection in marine reserves can support population resilience to large scale environmental impacts through maintenance of greater larval production and recruitment, stemming from combined contributions of the greater density and size of populations within reserves compared to fished populations outside reserves. Thus, we show that coastal marine reserves enhance the resilience of exploited populations through the multiplicative effect of large adult body size on reproductive output and local recruitment, two key components of population resilience, e.g. [29].

Our study elucidates the biological mechanism that may underlie resilience to climatic impacts in reserves. Increased abundance and broader size structure are commonly documented responses to local protection [14]. At Isla Natividad, the high mortality documented in 2009–2010 similarly affected individuals in different size classes, both within and outside the reserves. However, the absence of fishing mortality within reserves maintained slightly greater densities and sizes of protected populations. The effects of protection in reserves on egg production have been rarely assessed, compared to responses in terms of population abundance and size structure, but significant increase in egg production in reserves has also been documented in multiple marine organisms [30]–[32]. Thus, the mechanism we elucidated – increased or maintained reproduction and recruitment within and around reserves, relative to fished conditions - may underlie resilience in a broad suite of species and ecosystem types.

This work was conducted in coastal communities of Baja California, Mexico, that depend exclusively on the marine nearshore species that may be affected by the hypoxic zones that have recently developed along the western coast of north America [25]–[27]. Local livelihoods are threatened by these events, but our results show that the communities’ exclusive access rights to coastal resources and thus their capacity to establish and enforce marine reserves is effective in combating these unprecedented events. This context makes these results especially relevant to coastal communities and ecosystems of the Pacific and other worldwide locations similarly affected by regional and global change.

Recent research suggests that oxygen is declining globally in the oceans; midwater Oxygen Minimum Zones (OMZ) are expanding [33]–[35] and shoaling [36], with oxygen declines of >20% being observed in subthermocline waters [36]–[37]. Models and analyses of long-term data suggest that recent expansion of the OMZ and occurrence of hypoxic conditions along open coasts may be associated with climate change [33]–[34], [38].

In the California Current region, a range of natural processes, including episodic intrusions of deep water, seasonal upwelling, recent trends of shoaling OMZ’s, and the influence of Pacific Decadal Oscillation and El Nino Southern Oscillation cycles can all contribute to variability in hypoxic conditions on the shelf [27], [39]. This issue is particularly pertinent to the California Current compared to other upwelling ecosystems because the Eastern Pacific Ocean contains the world’s largest midwater OMZ which impinges on the continental margin [40]. Oxygen declines of >20% have been observed at 200–300 m depth off southern California [36]–[37], and the hypoxic boundary off Southern California has shoaled by at least 90 m in the past two decades [36].

Trends of expanding low-oxygen zones [36]–[37] and recent occurrences of coastal hypoxia across the California Current [25]–[27], combined with our documentation of hypoxic conditions at Isla Natividad in 2010 and measurements indicating hypoxic conditions in 2009 south of Isla Natividad (see *Introduction*) suggest that invertebrate mortality may have been associated with climate-induced hypoxia, though this hypothesis remains to be tested with continued monitoring and experiments. Regardless of the cause of the mortality events, our results indicate that protection in marine reserves supports resilience to sudden population reduction.

The limited spatial extent (<300 m) of the positive effect of reserves on recruitment suggests that a possible contribution of enhanced reproduction to fisheries catches, through larval spillover, may occur only in areas immediately adjacent to reserves. Models indicate that for this and other marine species characterized by limited dispersal, rebuilding depleted populations and fisheries will require the establishment of networks of multiple small marine reserves [41]–[42].

These data contribute new evidence to the active debate of whether ameliorating local disturbance (e.g., from fishing) is an effective strategy for addressing regional and global threats [3]–[4], [9], [13], [16], [18]–[20]. Our results indicate that management actions aimed at alleviating local stressors, such as protection in marine reserves, can increase the ability of populations to resist climate disturbances through maintained reproductive output (*resistance*), thereby preventing local extirpation, and possibly also their ability to reverse the effects of such disturbances (*recovery*) [3].

After the mass mortality events, the reserves established by the cooperative at Isla Natividad constitute the most productive sources of larvae and arguably can enhance local population recovery, though future recovery may depend on the frequency and severity of possible additional mortality events. Moreover, chronic stressors from increased mean temperatures and ocean acidification will further impact species that are vulnerable to these stressors. Under future scenarios of frequent and/or persistent disturbance [43], increasing resilience to climatic impacts through networks of marine reserves may be the most effective tool that local communities and nations worldwide have to combat the negative impacts of global climate change on marine ecosystems and livelihoods.

## Materials and Methods

### Field Monitoring

Measurements of dissolved oxygen (DO) concentrations and temperature were recorded every 15 minutes using autonomous sensors (Aanderaa Oxygen Optode 3835, Aanderaa Data Instruments) affixed onto moorings deployed at three sites around the island on 5 May 2010 at depths between 11.5–14.7 m and 550–2,700 m from the shore. The locations were selected to represent varying oceanographic conditions around Isla Natividad (see Fig. 1): (1) Morro Prieto, on the west side of the island, is typified by colder waters and consistent upwelling; (2) La Dulce, located in the Dewey Canal, experiences variable water temperatures; and 3) La Plana, located to the south of the island, is slightly shallower than the other two locations (11.5 m depth vs 14.7 m). Sensors were retrieved every two months to download the recorded data (Fig. S1).

Ecological monitoring was conducted yearly, in July-August, between 2006–2010 within the two no-take reserves and at three reference, fished sites (Fig. 1). Reference sites were selected among potential candidate sites to present similar conditions to reserves, based on initial surveys and analyses of benthic habitat and community characteristics (bottom rugosity, proportion of different substrate types, and the composition and structure of benthic communities). Reserves were established on the north-eastern (the Punta Prieta reserve, 0.8 km^2^) and south-eastern sides of the island (the La Plana-Las Cuevas reserve, 1.4 km^2^). Surveillance and enforcement of the no-take reserves is conducted by members of the fishing cooperative, as part of their continuous surveillance of their fishing grounds to prevent poaching. Thus, no illegal take occurred within reserves either by community members or other fishers. Abalone densities were estimated *in situ*, using scuba, within replicate 30×2 m belt transects laid haphazardly on the rocky bottom between 3–21 m depth. Between 11–30 transects were surveyed at each site, in each year (av. = 19.8, SE = 1.1). Significance of density variation between reserves and fished sites, and through time, was assessed with analysis of covariance (ANCOVA) conducted on [log(x+1)]-transformed density estimates from each transect. Protection (reserve vs. fished) and year (2006 through 2010) were fixed factors in the ANCOVA model, and site was a random factor, nested in protection. Mean depth of each transect was included as a covariate (Table S1). The size (maximum shell length, in cm) of pink abalones was recorded at the same sites and time of the year during replicate timed searches (lasting 25–70 min each, av. = 50 min, SE = 3 min) conducted by experienced local divers. Between 3–9 timed searches were conducted at each site, in each year (av. = 5.6, SE = 0.4). Kolmogorov-Smirnov tests were performed to examine differences in the size distributions between reserves and fished areas, in each year of the study (Fig. S2; Table S2).

### Estimates of Reproductive Output

Reproductive output, *R*, defined as the total number of eggs produced per unit area, was calculated in each year and for each of the two reserves and three fished areas, using the equation:
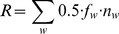
where 0.5 is the sex ratio, *f_w_* is the fecundity on individuals of weight *w* and *n_w_* is the mean density (ind m^−2^) of individuals (estimated through the belt transects) of weight *w* in the given year (derived from the individuals’ lengths, see below, which in turn were measured during the timed searches).

Fecundity in abalone is known to increase linearly with body mass [44]–[46]. The relationship between shell length (mm) and body mass (g) for pink abalone was obtained from [47]:
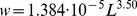
where *w* is the weight (g) and *L* is shell length (mm).

We assumed that the number of eggs produced by each female is zero below the size at sexual maturity (103.5 mm, corresponding to 156 g) [47]. Above the size at sexual maturity, we assumed a mean number of eggs per gram of female body weight of 2963 [the average between the reported values of 2078 and 3848] [44]:
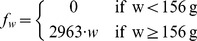



We bootstrapped the size frequency distributions and density estimates inside and outside the reserves in each year 10,000 times to evaluate the uncertainty in our estimation of reproductive output. The mean and the standard deviation of the reproductive output were computed on the 10,000 bootstrapped replicates (Table S3). Estimates of reproductive output in the reserves and in fished areas were then compared by using a randomization test performed on the bootstrapped distributions. We used the following procedure: (1) we randomly selected a value *θ_i_* of reproductive output from the bootstrapped distribution obtained for the reserves; (2) we randomly selected a second value *θ_k_* from the bootstrapped distribution obtained for fished areas; (3) we computed the difference between *θ_i_* and *θ_k_*. This procedure was repeated 1,000 times. Significance levels *P* were computed as the fraction of times the difference between *θ_i_* and *θ_k_* was positive (Table S3).

### Quantification of Larval Recruitment

To quantify and examine variation in abalone recruitment between reserves and fished sites, and at increasing distances from the edge of reserves, we deployed postlarval collectors at our field sites in 2008 and 2009. In both years, collectors were deployed during the abalone spawning season, October-January [48]. In 2008, we utilized two types of collectors to test the efficacy of different designs for collecting post-larval abalone recruits. The first collector design consisted of two clear 0.25×0.25 m corrugated polycarbonate plates fixed together horizontally with a 1.5 cm space between the plates [49]. The plates were mounted on a PVC plate and moored 0.5 m above the seafloor using PVC tubing affixed to a 22 kg sand-filled plastic base. The second collector was modeled after a design developed by Dr. Craig Mundy, at the University of Tasmania (personal communications). This collector consisted of six black 0.25×0.25 m high density polyethylene (HDPE) plates stacked between two PVC plates. The plates were cut from highly rugose three-dimensional sheets originally used for landscape drainage systems. The second collector type had the same mooring configuration as the transparent plates but was slightly shorter, extending 0.3 m from the seafloor.

In 2008, the two types of collectors were deployed within 2 m of each other at four locations within the Punta Prieta reserve and four locations within a reference site, between 2–3 km south-east of the reserve edge. Recruitment rates did not differ significantly between the two types of collector (ANOVA, *P* = 0.49). Based on this result, we used only the clear plastic collectors in 2009. In 2009, collectors were deployed, in pairs, at 4 and 6 locations within the Punta Prieta and the La Plana-Las Cuevas reserve, respectively, and at 4 and 3 locations within two fished areas. In addition, pairs of collectors were deployed at locations 150 and 300 m to the southeast of the Punta Prieta reserve, downstream from the reserve based on the dominant current direction. This design allowed for an evaluation of spatial patterns of recruitment from the reserve and away from its edges, at ∼150, 300, 1000, 1500, 2000 and 2500 m from its southern edge (Fig. S3). A total of 16 collectors were deployed in 2008, at depths ranging 9–14 m (avg. = 10.8 m, SE = 0.3), and 32 in 2009, at depths ranging 5–15 m (avg. = 9.9 m, SE = 0.6).

Before deployment, the plates were conditioned in 1 mm filtered seawater for a minimum of 10 days to grow a diatom film suitable for inducing post-larval settlement of abalone [49]–[50]. In both years, the plates were retrieved and replaced with conditioned plates approximately every two weeks. This two-week time interval was determined to be optimal for detecting post-larval settlement of abalone on collectors by [49]. Collectors were first deployed on 20 November 2008, followed by three subsequent plate exchanges. Final retrieval of collector plates occurred on 13 January 2009. In 2009, collectors were first deployed on 23 September, and plates were exchanged 5 times thereafter. Final retrieval occurred on 6 December 2009.

Collector plates were processed following the methodology employed by Nash et al. [51]. Samples were sieved through a 125 µm mesh, stained with a 0.5% Alizarin Red solution to facilitate sorting of samples, and preserved in 95% ethanol. Abalone postlarvae were subsequently identified and counted under a dissecting microscope. Abalone recruit abundances were analyzed using analyses of variance (ANOVAs). To compare recruitment rates between the two years, we performed ANOVAs with year (2008 and 2009) and protection (reserve vs. fished) as fixed, crossed factors, and date of collector retrieval as a random factor, nested within year (Table S4). Collectors were the replicate sampling units. To examine the effects of protection (in the two reserves) on recruitment rates in 2009, we used ANOVAs with protection (reserve vs. fished) as the fixed factor, site as a random factor, nested within protection, and date as a random, crossed factor (Table S5). Finally, recruitment variation with distance from the reserve was analyzed with ANOVAs including distance as a fixed factor (within the reserve, and at 150, 300, 1000, 1500, 2000, and 2500 m from its edge), and date as a random factor (Table S6). Initial models including collector location as an additional random factor showed that the effect of location was never significant (*P*>0.25 in all cases), and this factor was not included in subsequent models. Recruit abundances were transformed [log(x)+1] before ANOVAs.

## Supporting Information

Figure S1 **Mean daily average dissolved oxygen (DO) concentration at the three sites where sensors were deployed (La Plana, Morro Prieto, and La Dulce,** **Fig. 1** **).** Water depth is 11.5 m at La Plana, and 14.7 m at the other sites. Data were recorded at 15-min. intervals between 5 May-31 December 2010.
(DOCX)

Figure S2 **Size frequency distribution (% individuals in each size class) of pink abalones within reserves and fished areas.** Maximum shell lengths (cm) were binned in 2-cm intervals (the upper limit of each size bin is reported on the horizontal axis). The total number of individuals measured in each year is reported in each panel, as well as *P* values from Kolmogorov-Smirnov tests comparing size structure between reserves and fishes areas (Table S2).
(EPS)

Figure S3 **Location of recruitment collectors in the 2009 experiment.**
(DOCX)

Table S1 **Results of ANCOVA examining variation in pink abalone densities with protection (pr) and through time (ye).** Densities, estimated through belt transects, were [log(x+1)]-transformed Mean depth of transects (de) was included as a covariate.
(DOCX)

Table S2 **Results of Kolmogorov-Smirnov tests comparing size structure of pink abalones between years, and between reserves and reference, fished areas.** Significance of each pairwise comparison is reported. NS: not significant; *P = 0.05; *P<0.05; **P<0.01; ***P<0.001.
(DOCX)

Table S3 **Mean and standard deviation (** ***SD*** **) of 10,000 bootstrapped reproductive output estimates (No. eggs m^−2^ year^−1^), and significance levels obtained through the randomization test.**
(DOCX)

Table S4 **ANOVA testing variation in recruitment rates (No. abalone recruits/collector/2 weeks) between years (2008 and 2009, before and after the invertebrate mortality event) and protection level (the Punta Prieta marine reserve and a fished area located ∼2–3 km to the southeast of the reserve;** **Fig. 1** **).** Date of collectors’ retrieval was a random factor, nested within year.
(DOCX)

Table S5 **ANOVA testing variation in recruitment rates (No. abalone recruits/collector/2 weeks) in 2009 with protection level (reserves and fished areas).** Site (two reserves and two fished areas) is a random factor, nested within protection, and date of collector retrieval is random and crossed with the other factors.
(DOCX)

Table S6 **Results of ANOVA examining variation in recruitment rates (No. abalone recruits/collector/2 weeks) with distance from the reserve edge (di: within the reserve, and 150, 300, 1000, 1500, 2000 and 2500 m from its edge), and through time (da: 5 sampling dates).**
(DOC)
