# Supplementary figures and images for: Evidence That Marine Reserves Enhance Resilience to Climatic Impacts
Source: PLoS One. 2012 Jul 18;7(7):e40832. doi: 10.1371/journal.pone.0040832 (PMC3408031; doi:10.1371/journal.pone.0040832)

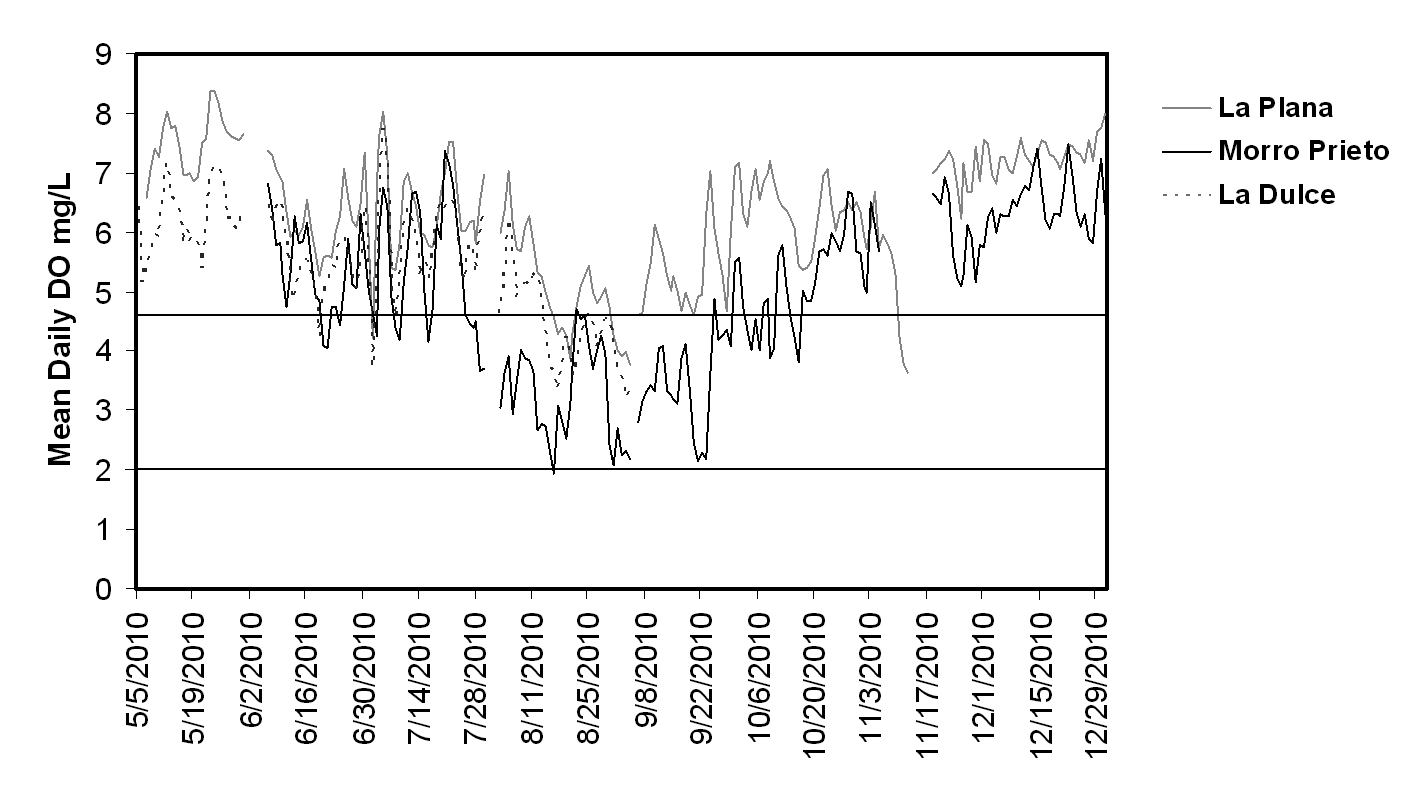

Supplement: Figure S1 — Mean daily average dissolved oxygen (DO) concentration at the three sites where sensors were deployed (La Plana, Morro Prieto, and La Dulce, Fig. 1 ). Water depth is 11.5 m at La Plana, and 14.7 m at the other sites. Data were recorded at 15-min. intervals between 5 May-31 December 2010. (DOCX) [file pone.0040832.s001.docx]

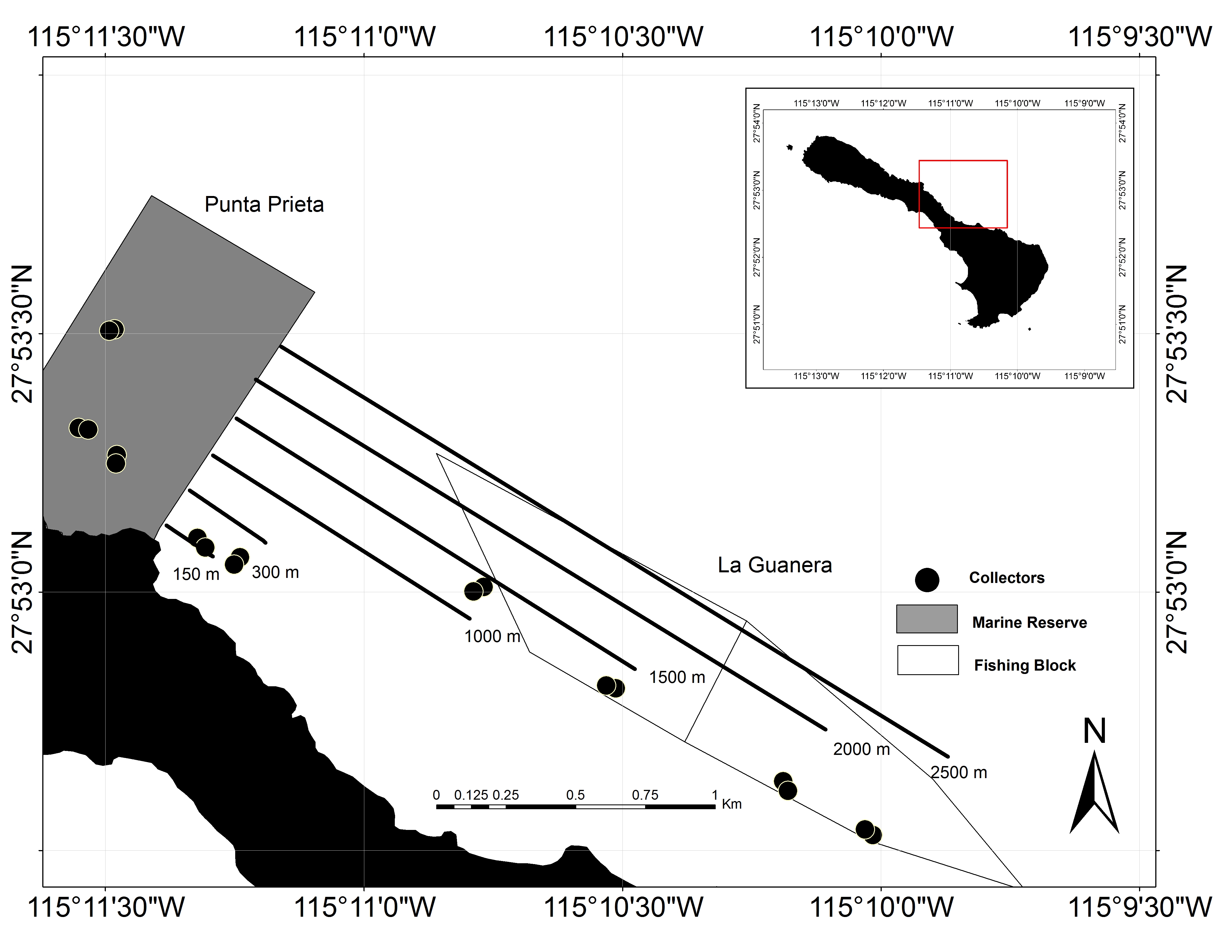

Supplement: Figure S3 — Location of recruitment collectors in the 2009 experiment. (DOCX) [file pone.0040832.s003.docx]
