# Supplementary material for: Evidence That Marine Reserves Enhance Resilience to Climatic Impacts
Source: PLoS One. 2012 Jul 18;7(7):e40832. doi: 10.1371/journal.pone.0040832 (PMC3408031; doi:10.1371/journal.pone.0040832)
Supplement: Table S1 — Results of ANCOVA examining variation in pink abalone densities with protection (pr) and through time (ye). Densities, estimated through belt transects, were [log(x+1)]-transformed Mean depth of transects (de) was included as a covariate. (DOCX) [file pone.0040832.s004.docx]

Source df SS MS *F* *P*

de 1 3.38 3.38 7.60 **0.006**

pr 1 6.5053E-2 6.5053E-2 0.15 0.89

ye 4 21.45 5.36 16.02 **0.001**

si(pr) 3 1.59 0.53 1.19 0.34

prxye 4 1.63 0.41 1.21 0.35

yexsi(pr) 11 3.68 0.33 0.75 0.69

Res 451 200.64 0.44
