# Supplementary material for: Evidence That Marine Reserves Enhance Resilience to Climatic Impacts
Source: PLoS One. 2012 Jul 18;7(7):e40832. doi: 10.1371/journal.pone.0040832 (PMC3408031; doi:10.1371/journal.pone.0040832)
Supplement: Table S2 — Results of Kolmogorov-Smirnov tests comparing size structure of pink abalones between years, and between reserves and reference, fished areas. Significance of each pairwise comparison is reported. NS: not significant; *P = 0.05; *P<0.05; **P<0.01; ***P<0.001. (DOCX) [file pone.0040832.s005.docx]

| **Reserve** |  | **Fished** |
| --- | --- | --- |
| 2006 | *** | 2006 |
| *** |  | *** |
| 2007 | NS | 2007 |
| * |  | *** |
| 2008 | ** | 2008 |
| NS |  | *** |
| 2009 | * | 2009 |
| NS |  | NS |
| 2010 | *’ | 2010 |
