# Supplementary material for: Evidence That Marine Reserves Enhance Resilience to Climatic Impacts
Source: PLoS One. 2012 Jul 18;7(7):e40832. doi: 10.1371/journal.pone.0040832 (PMC3408031; doi:10.1371/journal.pone.0040832)
Supplement: Table S3 — Mean and standard deviation ( SD ) of 10,000 bootstrapped reproductive output estimates (No. eggs m−2 year−1), and significance levels obtained through the randomization test. (DOCX) [file pone.0040832.s006.docx]

| **Year** | **Reserves** | | **Fished** | | ***P*** |
| --- | --- | --- | --- | --- | --- |
|  | Mean | SD | Mean | SD |  |
| 2006 | 8584.9 | 1648.8 | 9454.9 | 3354.20 | NS |
| 2007 | 24944.2 | 6022.3 | 20280.0 | 2887.4 | NS |
| 2008 | 23938.0 | 5048.5 | 17796.3 | 6204.7 | NS |
| 2009 | 14484.8 | 2283.4 | 8742.7 | 1658.0 | <0.05 |
| 2010 | 11835.4 | 5442.2 | 4485.3 | 910.5 | 0.05 |
