# Supplementary material for: Evidence That Marine Reserves Enhance Resilience to Climatic Impacts
Source: PLoS One. 2012 Jul 18;7(7):e40832. doi: 10.1371/journal.pone.0040832 (PMC3408031; doi:10.1371/journal.pone.0040832)
Supplement: Table S4 — ANOVA testing variation in recruitment rates (No. abalone recruits/collector/2 weeks) between years (2008 and 2009, before and after the invertebrate mortality event) and protection level (the Punta Prieta marine reserve and a fished area located ∼2–3 km to the southeast of the reserve; Fig. 1 ). Date of collectors’ retrieval was a random factor, nested within year. (DOCX) [file pone.0040832.s007.docx]

Source df SS MS *F* *P*

Ye 1 9.3732E-2 9.3732E-2 9.0631E-2 0.83

pr 1 3.68 3.68 6.19 **0.03**

da(ye) 7 7.24 1.03 4.26 **0.001**

yexpr 1 0.71 0.71 1.19 0.34

prxda(ye) 7 4.17 0.59 2.46 **0.02**

Res 96 23.28 0.24
