# Supplementary material for: Evidence That Marine Reserves Enhance Resilience to Climatic Impacts
Source: PLoS One. 2012 Jul 18;7(7):e40832. doi: 10.1371/journal.pone.0040832 (PMC3408031; doi:10.1371/journal.pone.0040832)
Supplement: Table S5 — ANOVA testing variation in recruitment rates (No. abalone recruits/collector/2 weeks) in 2009 with protection level (reserves and fished areas). Site (two reserves and two fished areas) is a random factor, nested within protection, and date of collector retrieval is random and crossed with the other factors. (DOCX) [file pone.0040832.s008.docx]

Source df SS MS *F* *P*

pr 1 3.58 3.58 10.76 **0.008**

da 4 1.40 0.35 0.74 0.56

si(pr) 2 0.68 0.34 0.73 0.51

prxda 4 0.15 3.6269E-2 7.7289E-2 0.99

si(pr)xda 8 3.77 0.47 2.17 **0.04**

Res 109 23.59 0.22
