# Supplementary material for: Evidence That Marine Reserves Enhance Resilience to Climatic Impacts
Source: PLoS One. 2012 Jul 18;7(7):e40832. doi: 10.1371/journal.pone.0040832 (PMC3408031; doi:10.1371/journal.pone.0040832)

**Marine reserves enhance resilience to climatic impacts**

Fiorenza Micheli.^1^, Andrea Saenz-Arroyo^2^, Ashley Greenley^1^, Leo Vazquez^2^, Jose Antonio Espinoza Montes^3^, Marisa Rossetto^4^, and Giulio A. De Leo^4^

*^1^Hopkins Marine Station, Stanford University, Pacific Grove, California, USA*

*^2^Comunidad y Biodiversidad A.C., Colonia Hipódromo Condesa, DF, México*

*^3^Sociedad Cooperativa de Produccion Pesquera Buzos y Pescadores, Isla Natividad, Baja California Sur, México*

*^4^Environmental Science Department, University of Parma, Parma, Italy*

**Supplementary Figures and Tables**

Table S1. Results of ANCOVA examining variation in pink abalone densities with protection (pr) and through time (ye). Densities, estimated through belt transects, were [log(x+1)]-transformed Mean depth of transects (de) was included as a covariate.

Source df SS MS *F* *P*

de 1 3.38 3.38 7.60 **0.006**

pr 1 6.5053E-2 6.5053E-2 0.15 0.89

ye 4 21.45 5.36 16.02 **0.001**

si(pr) 3 1.59 0.53 1.19 0.34

prxye 4 1.63 0.41 1.21 0.35

yexsi(pr) 11 3.68 0.33 0.75 0.69

Res 451 200.64 0.44

Table S2. Results of Kolmogorov-Smirnov tests comparing size structure of pink abalones between years, and between reserves and reference, fished areas. Significance of each pairwise comparison is reported. NS: not significant; *’ *P*=0.05; * *P*<0.05; ** *P*<0.01; *** *P*<0.001.

| **Reserve** |  | **Fished** |
| --- | --- | --- |
| 2006 | *** | 2006 |
| *** |  | *** |
| 2007 | NS | 2007 |
| * |  | *** |
| 2008 | ** | 2008 |
| NS |  | *** |
| 2009 | * | 2009 |
| NS |  | NS |
| 2010 | *’ | 2010 |

Table S3. Mean and standard deviation (*SD*) of 10,000 bootstrapped reproductive output estimates (No. eggs m^-2^ year^-1^), and significance levels obtained through the randomization test.

| **Year** | **Reserves** | | **Fished** | | ***P*** |
| --- | --- | --- | --- | --- | --- |
|  | Mean | SD | Mean | SD |  |
| 2006 | 8584.9 | 1648.8 | 9454.9 | 3354.20 | NS |
| 2007 | 24944.2 | 6022.3 | 20280.0 | 2887.4 | NS |
| 2008 | 23938.0 | 5048.5 | 17796.3 | 6204.7 | NS |
| 2009 | 14484.8 | 2283.4 | 8742.7 | 1658.0 | <0.05 |
| 2010 | 11835.4 | 5442.2 | 4485.3 | 910.5 | 0.05 |

Table S4. ANOVA testing variation in recruitment rates (No. abalone recruits/collector/2 weeks) between years (2008 and 2009, before and after the invertebrate mortality event) and protection level (the Punta Prieta marine reserve and a fished area located ~ 2-3 km to the southeast of the reserve; Fig. S1). Date of collectors’ retrieval was a random factor, nested within year.

Source df SS MS *F* *P*

Ye 1 9.3732E-2 9.3732E-2 9.0631E-2 0.83

pr 1 3.68 3.68 6.19 **0.03**

da(ye) 7 7.24 1.03 4.26 **0.001**

yexpr 1 0.71 0.71 1.19 0.34

prxda(ye) 7 4.17 0.59 2.46 **0.02**

Res 96 23.28 0.24

Table S5. ANOVA testing variation in recruitment rates (No. abalone recruits/collector/2 weeks) in 2009 with protection level (reserves and fished areas). Site (two reserves and two fished areas) is a random factor, nested within protection, and date of collector retrieval is random and crossed with the other factors.

Source df SS MS *F* *P*

pr 1 3.58 3.58 10.76 **0.008**

da 4 1.40 0.35 0.74 0.56

si(pr) 2 0.68 0.34 0.73 0.51

prxda 4 0.15 3.6269E-2 7.7289E-2 0.99

si(pr)xda 8 3.77 0.47 2.17 **0.04**

Res 109 23.59 0.22

Table S6. Results of ANOVA examining variation in recruitment rates (No. abalone recruits/collector/2 weeks) with distance from the reserve edge (di: within the reserve, and 150, 300, 1000, 1500, 2000 and 2500 m from its edge), and through time (da: 5 sampling dates).

Source df SS MS *F* *P*

di 6 7.39 1.23 2.97 **0.03**

da 4 1.97 0.49 0.91 0.47

dixda 24 9.95 0.41 0.77 0.73

Res 55 29.81 0.54

Figure S1. Mean daily average dissolved oxygen (DO) concentration at the three sites where sensors were deployed (La Plana, Morro Prieto, and La Dulce). Water depth is 11.5 m at La Plana, and 14.7 m at the other sites. Data were recorded at 15-min. intervals between 5 May-31 December 2010.


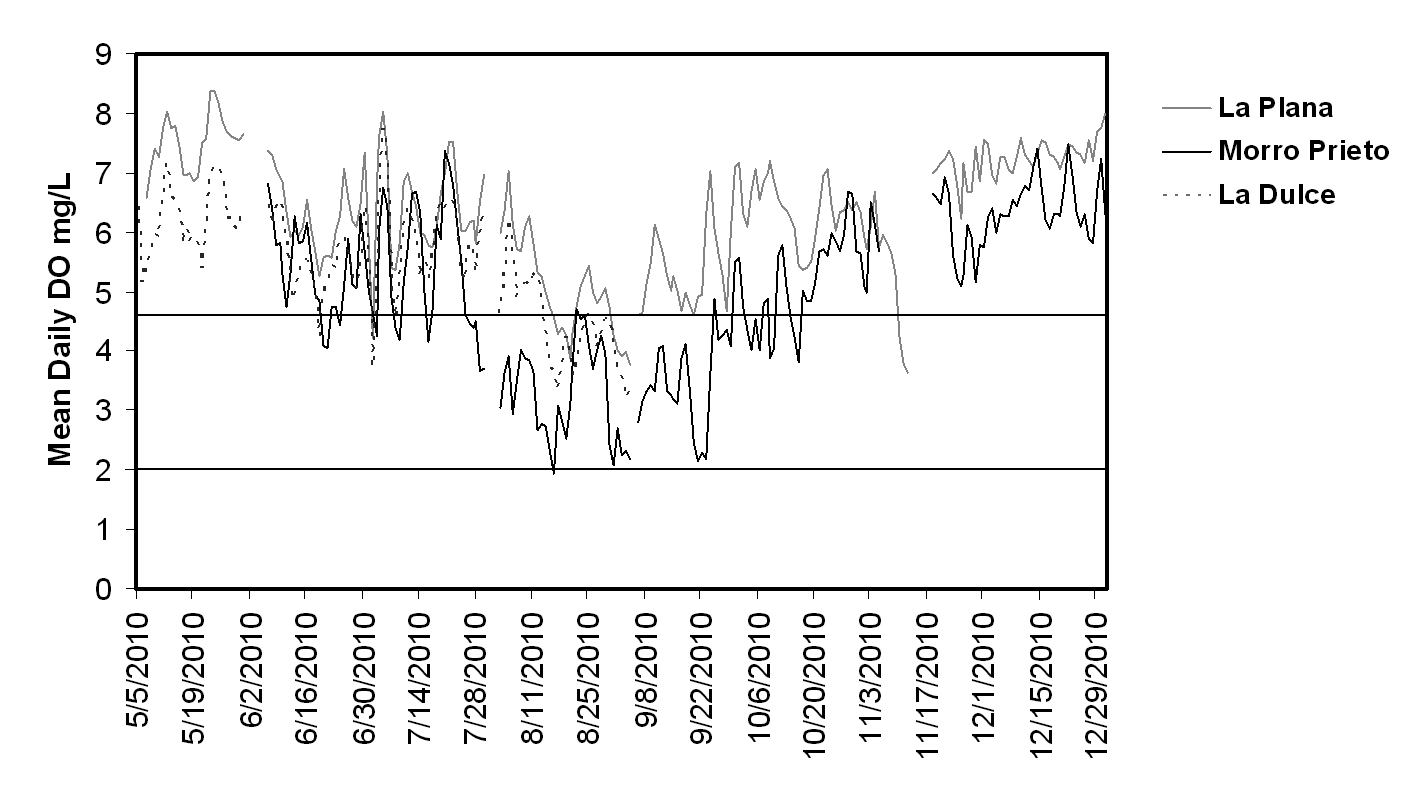


Figure S2. Size frequency distribution (% individuals in each size class) of pink abalones within reserves and fished areas. Maximum shell lengths (cm) were binned in 2-cm intervals (the upper limit of each size bin is reported on the horizontal axis). The total number of individuals measured in each year is reported in each panel, as well as *P* values from Kolmogorov-Smirnov tests comparing size structure between reserves and fishes areas (Table S2).


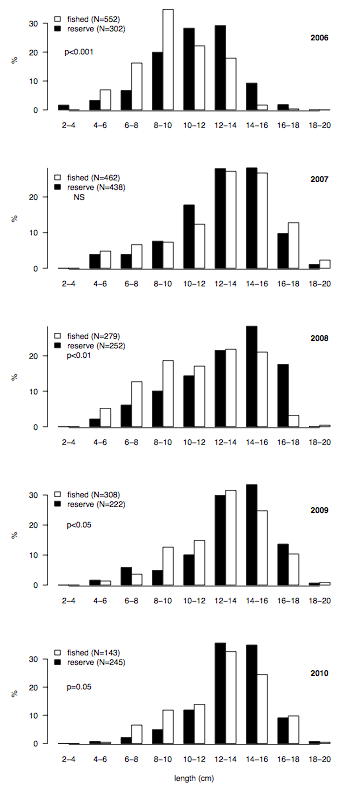


Figure S3. Location of recruitment collectors in the 2009 experiment.


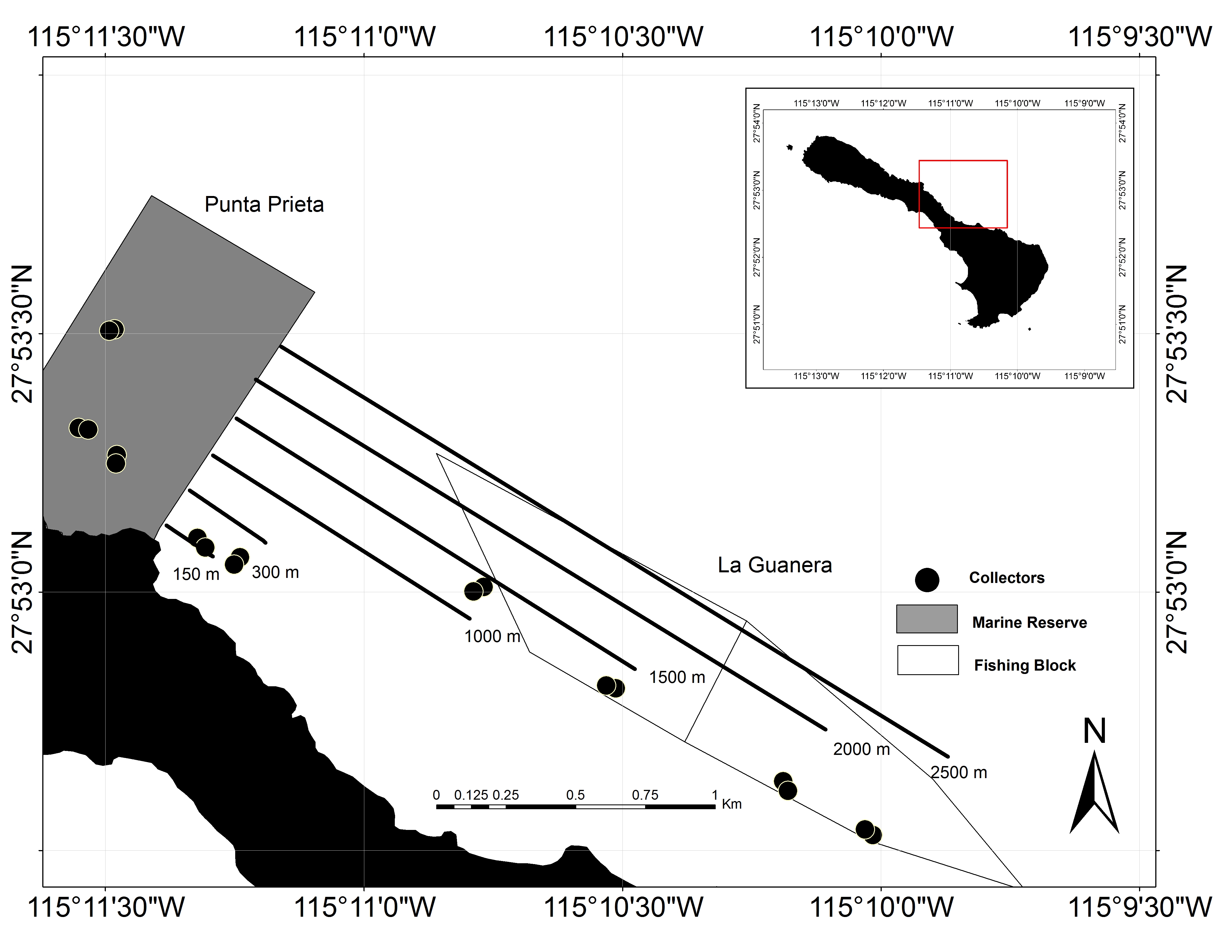

Supplement: Table S6 — Results of ANOVA examining variation in recruitment rates (No. abalone recruits/collector/2 weeks) with distance from the reserve edge (di: within the reserve, and 150, 300, 1000, 1500, 2000 and 2500 m from its edge), and through time (da: 5 sampling dates). (DOC) [file pone.0040832.s009.doc]
